# UPDATE - 2022 Italian guidelines on the management of bronchiolitis in infants

**DOI:** 10.1186/s13052-022-01392-6

**Published:** 2023-02-10

**Authors:** Sara Manti, Annamaria Staiano, Luigi Orfeo, Fabio Midulla, Gian Luigi Marseglia, Chiara Ghizzi, Stefania Zampogna, Virgilio Paolo Carnielli, Silvia Favilli, Martino Ruggieri, Domenico Perri, Giuseppe Di Mauro, Guido Castelli Gattinara, Antonio D’Avino, Paolo Becherucci, Arcangelo Prete, Giuseppe Zampino, Marcello Lanari, Paolo Biban, Paolo Manzoni, Susanna Esposito, Giovanni Corsello, Eugenio Baraldi

**Affiliations:** 1 University of Messina, Messina, Italy; 2 SIP “Società Italiana di Pediatria”, University “Federico II”, Naples, Italy; 3 SIN “Società Italiana di Neonatologia”, Hospital San Giovanni Calibita Fatebenefratelli, Rome, Italy; 4 SIMRI ”Società Italiana per le Malattie Respiratorie Infantili”, University of Rome “La Sapienza”, Rome, Italy; 5 SIAIP “Società Italiana di Allergologia e Immunologia Pediatrica”, Foundation IRCCS Policlinico San Matteo, Pavia, Italy; 6 AMIETIP ”Accademia Medica Infermieristica di Emergenza e Terapia Intensiva Pediatrica”, Major Hospital Polyclinic: Fondazione IRCCS Ca’ Granda Ospedale Maggiore Policlinico, Bologna, Italy; 7 SIMEUP “Società Italiana di Medicina di Emergenza ed Urgenza Pediatrica”, Pugliese Ciaccio Hospital, Catanzaro, Italy; 8 SIMP “Società Italiana di Medicina Perinatale”, University Hospital of Ancona Umberto I G M Lancisi G Salesi, Ancona, Italy; 9 SICP “Società Italiana di Cardiologia Pediatrica”, University Hospital Meyer, Firenze, Italy; 10 SINP “Società Italiana di Neurologia Pediatrica”, University of Catania, Catania, Italy; 11 SIPO “Società Italiana Pediatria Ospedaliera”, San Giuseppe Moscati Hospital, Aversa, Italy; 12 SIPPS “Società Italiana di Pediatria Preventiva e Sociale”, Local Health Authority Caserta, Caserta, Italy; 13 SITIP “Società Italiana di Infettivologia Pediatrica”, Bambino Gesu Pediatric Hospital, Rome, Italy; 14 FIMP “Federazione Italiana Medici Pediatri”, Local Health Authority Naples 1 Centre, Naples, Italy; 15 SICuPP “Società Italiana delle Cure Primarie Pediatriche”, Florence City Council, Florence, Italy; 16 AIEOP “Società Italiana di Ematologia e Oncologia Pediatrica”, IRCCS University Hospital of Bologna, Bologna, Italy; 17 SIMGePeD “Società Italiana Malattie Genetiche Pediatriche e Disabilità Congenite”, University Hospital Agostino Gemelli, Rome, Italy; 18 Alma Mater Studiorum University of Bologna, Bologna, Italy; 19 University Hospital of Verona, Verona, Italy; 20 Ospedale Degli Infermi, Biella, Italy; 21 University of Turin, Turin, Italy; 22 University of Parma, Parma, Italy; 23 University of Palermo, Palermo, Italy; 24 Department of Women’s and Children’s Health, Neonatal Intensive Care Unit, University Hospital of Padova, Padova, Italy

**Keywords:** Bronchiolitis, Guidelines, Infants, Respiratory syncytial virus, Update, Prevention

## Abstract

Bronchiolitis is an acute respiratory illness that is the leading cause of hospitalization in young children. This document aims to update the consensus document published in 2014 to provide guidance on the current best practices for managing bronchiolitis in infants**.** The document addresses care in both hospitals and primary care. The diagnosis of bronchiolitis is based on the clinical history and physical examination. The mainstays of management are largely supportive, consisting of fluid management and respiratory support. Evidence suggests no benefit with the use of salbutamol, glucocorticosteroids and antibiotics with potential risk of harm. Because of the lack of effective treatment, the reduction of morbidity must rely on preventive measures. De-implementation of non-evidence-based interventions is a major goal, and educational interventions for clinicians should be carried out to promote high-value care of infants with bronchiolitis. Well-prepared implementation strategies to standardize care and improve the quality of care are needed to promote adherence to guidelines and discourage non-evidence-based attitudes. In parallel, parents' education will help reduce patient pressure and contribute to inappropriate prescriptions. Infants with pre-existing risk factors (i.e., prematurity, bronchopulmonary dysplasia, congenital heart diseases, immunodeficiency, neuromuscular diseases, cystic fibrosis, Down syndrome) present a significant risk of severe bronchiolitis and should be carefully assessed. This revised document, based on international and national scientific evidence, reinforces the current recommendations and integrates the recent advances for optimal care and prevention of acute bronchiolitis.

**Supplementary Information:**

The online version contains supplementary material available at 10.1186/s13052-022-01392-6.

## Introduction

Viral bronchiolitis is the most frequent lower respiratory tract infection and the leading cause of hospitalization and death in infants less than twelve months of age [1]. Clinically, infants with bronchiolitis may experience a wide range of non-specific signs and symptoms, ranging from mild to severe respiratory distress and potentially culminating in acute respiratory failure [1].

The most common causative agent is *Respiratory Syncytial Virus* (RSV); it has been estimated that RSV infects more than 60% of all children during the first year of life, and that RSV infects nearly all children by the time they are 2 years old [2, 3]. *Rhinovirus* (RV), *Parainfluenza virus*, *Metapneumovirus* (MPV), *Influenza virus*, and *Adenovirus*, alone or in the form of co-infection, have also been reported [4].

The estimated global impact of RSV-caused infections in infants younger than 5 years of age was reported being approximately 33 million (range: 21.6–50.3 million), with 3.2 million hospitalizations (range: 2.7–3.8 million), and 120,000 deaths (range: 94,000–149,000) annually [5, 6]. A rising intensity of care for children with bronchiolitis with increased intensive care admissions has been observed worldwide in the last years [7, 8].

To date, due to the coronavirus disease of 2019 (COVID-19) pandemic, drastic changes in the epidemic curve of RSV have been reported. The state-mandated COVID-19 public health measures, particularly lockdowns and school closures, social distancing, hand washing, and masking, had led first to a drastic reduction in the number of cases of bronchiolitis worldwide and second to the resurgence of RSV when such measures had been lifted, ultimately disrupting the routine, historical seasonality with subsequent peaks in atypical periods of the year – thus, leading to a considerable impact on the healthcare systems worldwide [9–12].

Despite numerous clinical practice guidelines, there is tremendous variation in approaches to diagnosis, monitoring, and managing viral bronchiolitis [13]. It is, therefore, essential to unify diagnostic and therapeutic criteria.

Globally, the diagnosis of bronchiolitis is based on the clinical history and physical examination. Since a specific etiological treatment is unavailable, bronchiolitis therapy includes general supportive management to control pulmonary and systemic clinical symptoms. Moreover, due to the lack of a vaccine against RSV, environmental measures are crucial in preventing and limiting bronchiolitis spreading. Pharmacological immunoprophylaxis has proven beneficial to populations at increased risk for RSV infection–related complications, such as preterm babies [1], and it is currently licensed and available only for these specific infants.

This intersociety consensus document aims to update the clinical practice guideline entitled “Inter-society consensus document on treatment and prevention of bronchiolitis in newborns and infants”, published in 2014 [1]. By incorporating new evidence, this intersociety consensus document provides physicians, nurses and pediatric healthcare providers the recommendations to guide the diagnosis, management, treatment and prevention of infants with bronchiolitis. The document addresses care in both hospitals and primary care. The guidelines apply to children in the first twelve months of age.

The workgroup that performed the necessary literature research in drafting this recommendation document used the PubMed, EMBASE and Global Health databases (up to April 2022).

Each key action statement is associated with the level of evidence and recommendation and the benefit-harm relationship.

## Methods

In April 2021, a national working group convened a new committee to review the 2014 bronchiolitis guidelines [1]. The committee included general paediatricians and pediatric subspecialists, specifically hospitalists, pulmonologists, emergency physicians, neonatologists, pediatric infectious disease physicians and primary care paediatricians. The Italian Working Group started from both a critical appraisal of the literature and the evidence from the previous inter-society consensus document [1].

**Fig. 1:**
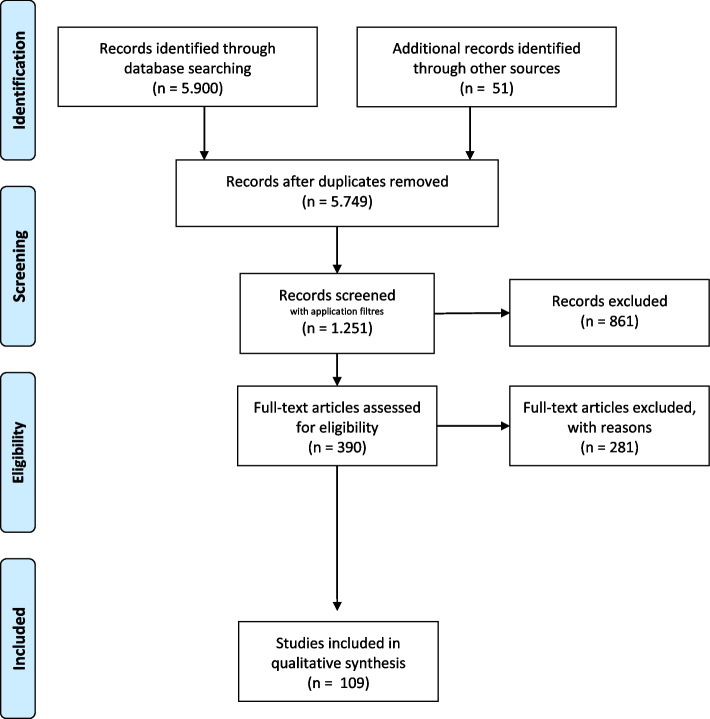
Flow chart of the literature research for two independent reviewers

The evidence search and review included electronic databases, including PubMed, EMBASE, and Global Health, and manuscripts published between October 2014 and April 2022. The search strategy is detailed in Additional file 1: Appendix 1. Manual searches of grey literature and guideline-focused databases/repositories [Web of Science, Google Scholar, BMJ Best Practice, National Institute for Health and Care Excellence, and World Health Organization (WHO)] were conducted using variations on terms for “bronchiolitis”, “acute”, “viral”, “newborn”, “infant” “children”, “child”, “RSV”, and “Respiratory Syncytial Virus”. In addition, selected references of included papers were searched to identify other relevant documents for inclusion. The references were regularly updated during the drafting of the guidelines. The Prisma [14] flow diagram of the search strategy is reported in Fig. 1.

The panel followed a systematic process that included a standardised methodology for rating the certainty of the evidence and strength of recommendation using the Grading of Recommendations, Assessment, Development and Evaluations (GRADE) methodology [15]. The following tools for evaluating the validity of the studies analysed were adopted: Grilli criteria for Consensus and Position Papers [16], the AGREE II checklist for guidelines ([17], Additional file 1: Appendix 2, Table 1), the AMSTAR-2 for the systematic review [18], the Newcastle Ottawa Scale-checklists for observational studies (e.g., cohort, case-control and cross-sectional studies) [19]. Clinical questions identifying Population (P), Intervention (I), Comparison (C) and Outcome (O) (P.I.C.O.) were addressed by methodologists and defined as follows:

1. Population

**Table 1:** AGREE II domain scores for included guidelines

Guideline (Year)	Domain					
	Scope and purpose	Stakeholder involvement	Rigour of development	Clarity of presentation	Applicability	Editorial independence
ISCD (2014) [1]	64%	52%	40%	54%	14%	42%
AAP (2014) [20]	90%	80%	91%	91%	42%	100%
JSPID (2017) [21]	20%	16%	34%	45%	0%	0%
ABG (2019) [22]	87%	66%	78%	87%	64%	35%
MSPBS (2016) [23]	29%	15%	0%	74%	3%	1%
SAP (2015) [24]	71%	21%	17%	85%	44%	0%
NICE (2015) [25]	91%	80%	90%	93%	95%	91%
IMSS (2015) [26]	86%	66%	57%	92%	20%	66%
FMSD (2015) [27]	33%	26%	45%	54%	8%	3%
DGSP (2015) [28]	91%	43%	35%	83%	44%	36%
SGSSS (2014) [29]	99%	97%	75%	68%	32%	50%
JAID/JSC (2016) [30]	52%	54%	8%	36%	1%	24%
GOUH (2014) [31]	85%	33%	30%	85%	21%	38%
CPS (2014) [32]	80%	33%	22%	63%	26%	0%

Neonate(s), newborn(s), infant(s), child, children, bronchiolitis, acute bronchiolitis, viral bronchiolitis, respiratory viruses, RSV.

2. Intervention

Clinical score, nasopharyngeal swab, reverse transcription polymerase chain reaction (RT-PCR), antigen detection, laboratory tests, chest-X-ray, lung ultrasound, nasogastric feeding, nebulized saline solution, nebulized hypertonic saline solution, intravenous hydration, superficial nasal aspiration, deep nasal aspiration, oxygen saturation level at air ambient, pulse oximetry, oxygen therapy, respiratory support (High flow nasal cannula (HFNC), continuous positive airway pressure (CPAP), environmental prophylaxis, chest physiotherapy, inhaled therapy (bronchodilators, adrenaline, steroids, furosemide, ipratropium bromide, helium), systemic treatment (steroids, antibiotics, ribavirin, montelukast, DNase, magnesium sulfate, methylxanthine), environmental prophylaxis, pharmacological prophylaxis.

3. Comparison

Standard care or treatment as usual or an alternative intervention, no treatment.

4. Outcome

Disease course, duration of acute illness disease severity, symptom score, adverse events, complications, emergency medical services, rate of access to the emergency department, hospital admission rate, transfer to intensive care unit (ICU), need for respiratory support, length of hospital stay.

### Eligibility criteria

Inclusion criteria were: paediatric population; clinical practice guideline or guidance document with recommendations for the management of bronchiolitis produced by global or national bodies; and the most up-to-date versions of guidelines (if multiple iterations); review; observational study; clinical trials, meta-analysis or systematic reviews not part of clinical practice guidelines. Exclusion criteria were: case report, case series, and withdrawn or superseded guidelines.

### Manuscript review

Two independent reviewers (SM, EB) performed data extraction, using standard templates to report recommendations in support of or against each area, and noting whether manuscripts reported equivocal evidence.

A draft version of the document underwent extensive peer review by committee experts in the field. The committee reviewed the resulting comments and, if appropriate, incorporated them into the final draft. The subject was divided into seven main topics: diagnosis, paediatric primary health care and assistance, indications for hospitalization, indications to transfer to intensive care unit, treatment (treatments recommended based on evidence, treatments for which evidence is equivocal, treatments not recommended based on evidence), indications to discharge, and prevention. Each key action statement was associated with the level of evidence and recommendation and the benefit-harm relationship.

## Results

Based on the evidence obtained for each question, the recommendations were formulated. All questions answered on the management of bronchiolitis were summarized in Table 2.

**Table 2:** Questions answered in regards to the management of bronchiolitis

Number	Questions
1	How is bronchiolitis diagnosed?
2	What is the role of primary care paediatricians in managing a child with bronchiolitis?
3	Are laboratory (blood and/or urine) tests and radiological exams supported in managing bronchiolitis?
4	When making decisions about the hospitalization of an infant with bronchiolitis?
5	When making decisions to transfer to intensive care unit an infant with bronchiolitis?
6.1	Which are evidence-based treatment recommendations?
6.2	Evidence is equivocal for which treatment?
6.3	Which are treatments not recommended based on evidence?
7	What criteria should be used for safe discharge?
8	How can we prevent bronchiolitis?

### How is bronchiolitis diagnosed?

*Recommendations:* The diagnosis of bronchiolitis is based on the clinical history and physical examination (Evidence Quality: B; Recommendation Strength: Strong Recommendation).

The collection of data on clinical history must investigate the presence of the following: exposure to individuals presenting with upper respiratory tract viral infections during the epidemic season, underlying conditions that may be associated with an increased risk of progression to severe morbidity or mortality, e.g., in utero smoke exposure, prematurity, congenital anomalies, genetic abnormalities, haemodynamically significant congenital heart disease, chronic lung disease (bronchopulmonary dysplasia (BPD)), and the presence of an immunocompromising state [1, 20–32].

Combined with the collection of clinical history, the physician should look for a wide range of suggestive but not specific clinical symptoms such as rhinorrhea and/or upper respiratory tract infections; first episode of respiratory distress associated with cough; crackles and/or wheezing; dyspnea; polypnea; increased respiratory effort manifested as nasal flaring, grunting, use of accessory muscles or intercostal and/or subcostal chest wall retractions; low oxygen (O_2_) saturation levels, apnea; skin colour changes; feeding difficulties; lethargy; and fever [1, 20–32].

The peak severity of the disease occurs around 3-5 days from the disease onset, and improvement occurs in 7-14 days, with 90% of infants having a resolution of cough within 2-3 weeks [1, 20–32].

Several clinical scores have been proposed to guide treatment and resource allocation in acute bronchiolitis [33–35]. However, due to the weak relationship between changes in scores and the clinical picture and the high inter-rater variability among the physicians [1, 20–32], clinical scores should be used in conjunction with careful clinical evaluation to improve and individualize the decision-making process.

### What is the role of primary care paediatricians in managing a child with bronchiolitis?

*Recommendations:* Primary care paediatricians should educate family members on evidence-based prevention, diagnosis, and management of bronchiolitis (Evidence Quality: C; Recommendation Strength; Moderate Recommendation).

Most children with acute bronchiolitis may be adequately managed in the outpatient setting by primary care paediatricians, parents or caregivers able to provide assistance and monitoring. After having ascertained the parents compliance and the presence of any risk factors (Table 3), the clinician must educate the parents or caregivers on the following:

1. how to assess the child’s general clinical conditions;
2. which supportive therapies to administer (see the chapter “Treatment”); and
3. when to ask for primary care paediatricians or when to access the Emergency Room.

**Table 3:** Risk factors for severe bronchiolitis

• Infants born prematurely (<35 weeks’ gestation) • <3 months of age at presentation • Decreased hydration and feeding (<50% of usual fluid intake in preceding 24 h) • Hemodynamically significant cardiac disease • Chronic lung disease • Neurological disorders • Immunodeficiency • Environmental factors: exposure to tobacco smoke and or air pollution • Social factors: distance from the hospital or difficulty to access to the hospital; poor social circumstances; unreliable parents or parents do not able to spot red flag symptoms	

The following signs of a worsening condition should be promptly recognized: reduced feeding; increased respiratory rate; onset of laboured breathing suggested by flared nostrils; use of accessory muscles, retractions, cyanosis, apnea, fewer wet diapers, or a generally toxic appearance [1, 20–32].

Initiatives to reduce non-evidence-based treatments in bronchiolitis, such as using common electronic medical records with quality control of treatments, can contribute to avoiding the prescription of ineffective medicines.

In addition, the creation and diffusion of parents' educational materials (information leaflet) on the evidence-based management of bronchiolitis can inform parents who often expect and demand medications for their sick infants and avoid the "doctor-shopping" for treating bronchiolitis. In parallel, the diffusion of information leaflets about environmental prophylaxis of RSV and other viruses can be helpful in protecting infants from these infections [36, 37].

### Are laboratory (blood an/or urine) tests and radiological exams supported in managing bronchiolitis?

*Recommendations:* Neither laboratory tests nor radiological exams are usually indicated for the routine work-up of infants with bronchiolitis (Evidence Quality: B; Recommendation Strength: Moderate Recommendation).

Laboratory tests are not usually indicated for the routine work-up of infants with bronchiolitis [1, 20–32, 38]. Complete blood counts, serum electrolytes, blood gas analysis, urinalysis and urine culture should not be routinely performed [1, 20–32, 38]. A bacterial co-infection is rarely associated with bronchiolitis; thus, guidelines [1, 20–32, 38] recommend against complete blood counts and cultures unless there is clinical evidence or strong suspicion of sepsis. The clinical examination is considered sufficient to assess the hydration status; thus, the measurement of serum electrolytes is not supported [1, 20–32, 38]. Unless signs and symptoms of severe respiratory distress or respiratory failure occurr, the blood gas analysis should not be mandatorily performed ([1, 20–32, 38], Tables 4 and 5). Because performing urinalysis and urine culture could expose the patient to unnecessary procedures and inappropriate treatment, urinalysis and urine culture should not be routinely performed [1, 20–32, 38]. Similarly, the value of identifying a specific viral aetiology causing bronchiolitis has not been demonstrated. RSV testing (nasopharyngeal swab) can be considered in the hospital setting for cohorting, decreasing antibiotic use, and epidemiological surveillance. The traditional or real-time polymerase chain reaction (RT-PCR) remains the gold standard diagnostic test. However, it is more expensive and not always available than antigen detection (immunofluorescence, enzyme immunoassay).

**Table 4:** Bronchiolitis severity

	Mild	Moderate	Severe
**Respiratory rate**	Normal to slightly increased	Increase	Markedly increased compared to normal values per age range (< 2 months: <60/min) (2-12 months: <50/min)
**Respiratory effort**	Mild chest wall retraction	Tracheal tug Nasal Flare Moderate chest wall retraction	Marked chest wall retraction Nasal Flare Grunting
**Oxygen saturation**	No supplemental oxygen requirement, O_2_ saturation > 95%	O_2_ saturation 90-95%	O_2_ saturation < 90%, may not be corrected by O_2_
**Feeding**	Normal to slightly decreased	50-75% of normal feeds	< 50% of feeds, unable to feed
**Apnea**	Absent	May have brief episodes	May have increasing episodes

**Table 5:** Scoring of the acute bronchiolitis severity scale

Score	0	1	2	3	4
**Wheezing**	No	Wheezing at end of expiration	Wheezing throughout expiration	Wheezing during inspiration-expiration	Severe hypoventilation
**Crackles**	No	Crackles in 1 field	Crackles in 2 fields	Crackles in 3 fields	Crackles in 4 fields
**Effort**	No effort	Subcostal or lower intercostal retractions	+ Suprasternal retractions or nasal flaring	+ Nasal flaring and suprasternal retractions (universal)	
**I:E ratio**^**a**^	Normal	Symmetrical	Inverted		

^a^Inspiration-to-Expiration ratio

In line with the above recommendations, chest radiography is not routinely recommended and should be widely restricted, as it could expose the patient to unnecessary and harmful procedures. It has been estimated that infants with bronchiolitis undergoing chest radiography are 10 times more likely to receive antibiotics [39].

In this regard, lung ultrasound performed in a hospital setting has been demonstrated to help stratify the risk of bronchiolitis and predict respiratory failure and the need for invasive ventilation without the risks associated with ionising radiation [40]. Accordingly, lung ultrasound appeared to be a feasible tool that might help the physician to confirm the clinical impression, predict hospital admission, the bronchiolitis severity, the need for respiratory support, and the length of hospital stay [41–47]. However, multicenter studies are needed to determine its value in clinical routine, the most optimal setting, and the target population.

### When making decision about the hospitalization of an infant with bronchiolitis?

*Recommendations:* Moderate-to-severe bronchiolitis and well-known risk factors for developing severe bronchiolitis must be considered for hospital admission (Evidence Quality: B; Recommendation Strength: Moderate Recommendation).

When deciding whether to hospitalize, the physician should remember that acute bronchiolitis can be complicated by significant temporal variability in the disease state and require serial observations over time to check and re-check the progression of clinical signs [1, 20–32].

The decision to admit to the hospital should be based on: clinical conditions suggesting moderate-to-severe bronchiolitis (Table 4), ability to maintain adequate hydration, and uncertainty over the diagnosis of bronchiolitis [1, 20–32, 48]. O_2_ saturation levels persistently lower than 92% must also be considered a criterion for the hospital admission. However, different O_2_ saturation lower thresholds have been recommended for guiding hospital admission [48].

The well-known risk factors for developing severe bronchiolitis (i.e., prematurity, BPD, congenital heart diseases, immunodeficiency, neuromuscular disease, cystic fibrosis, and Down syndrome) must also be considered for the decision on hospital admission (Table 3). The reliability of parents or caregivers must also be taken into account.

### When making decision to transfer to intensive care unit an infant with bronchiolitis?

*Recommendations:* Infants with bronchiolitis and respiratory failure requiring ventilatory support, oxygen saturation (SO_2_) reduced despite O_2_ therapy and or HFNC, apnea with desaturation, and severe impairment of general conditions must be transferred to the ICU (Evidence Quality: B; Recommendation Strength: Moderate Recommendation).

Infants with acute bronchiolitis must be referred to the ICU when the following occur: respiratory failure requiring respiratory support (CPAP), apnea with desaturation, and severe impairment of general conditions [1, 20–32, 48].

An acute bronchiolitis severity score (ABSS) has been proposed to help decide ICU admission, but it still requires validation in clinical practice ([49], Table 5).

The most common characteristics of infants with bronchiolitis admitted to ICU are median age of 60 days, male gender, prematurity, low birth weight, tachypnea, pre-existing co-morbidities, RSV-caused bronchiolitis, and co-infections (RSV, RV and bacterial pathogens) [1, 20–32, 48].

### Treatment

Since a specific etiological treatment is not available, bronchiolitis therapy includes general supportive management to control pulmonary and systemic clinical symptoms [1, 20–32].

Therapy for bronchiolitis and related recommendations for clinical practice are summarized in Table 6. Inhaled bronchodilators, nebulized adrenaline, steroids (systemic or nebulized) and antibiotics are not recommended.

**Table 6:** Treatment for bronchiolitis

Treatment	Indications	Evidence Quality Recommendation Strength
*Supportive treatment*	Recommended	Evidence Quality: A Recommendation Strength: Strong
*Oxygen therapy*	Recommended *(when SpO*_*2*_*<92%)*	Evidence Quality: A Recommendation Strength: Strong
*HFNC*	Recommended when standard subnasal supplemental O2 fails in infants who are hypoxic. *(It should not be used as a primary treatment modality)*	Evidence Quality: B Recommendation Strength: Moderate
*Nebulized hypertonic saline solution*	Not Recommended	Evidence Quality: B Recommendation Strenght: Moderate
*Inhaled bronchodilators*	Not Recommended	Evidence Quality: B Recommendation Strength: Strong
*Chest physiotherapy*	Not Recommended	Evidence Quality: A Recommendation Strength: Moderate
*Nebulized adrenaline*	Not Recommended	Evidence Quality: B; Recommendation Strength: Strong
*Nebulized steroids*	Not Recommended	Evidence Quality: A Recommendation Strength: Strong
*Systemic steroids*	Not Recommended	Evidence Quality: A Recommendation Strength: Strong
*Antibiotics*	Not Recommended *(Except in case of strong suspicion or clear evidence of a secondary bacterial infection)*	Evidence Quality: B; Recommendation Strength: Strong
*Other* *Antivirals* *Montelukast* *DNase* *Inhaled furosemide* *Inhaled ipratropium bromide* *Magnesium sulfate* *Helium* *Surfactant* *Methylxanthine*	Not Recommended	Evidence Quality: B; Recommendation Strength: Strong

#### Which are evidence-based treatment recommendations?

##### Superficial nasal aspiration

*Recommendations:* A gentle, superficial and reasonably frequent nasal aspiration, especially in younger children, is recommended to improve airway patency, O_2_ saturation measured by pulse oximetry (SpO_2_), and feeding (Evidence Quality: A; Recommendation Strength: Strong Recommendation).

Nasal suctioning should be performed before measuring O_2_ saturation in infants with bronchiolitis to avoid the overdiagnosis of hypoxaemia. It should be performed especially in younger children to improve airway patency, SpO_2_, and feeding [1, 20–32, 38, 48, 50].

##### Oxygen therapy

*Recommendations:* Supplemental O_2_ should be administered if O_2_ saturation levels are persistently below 92% in room air (Evidence Quality: A; Recommendation Strength: Strong Recommendation).

The levels of O_2_ saturation used as a guide for starting supplemental O_2_ therapy ranges from <90% to <95% among guidelines [1, 20–32, 38, 51]. However, the most commonly recommended cut-off in Europe and Australasia is <92% [1, 20–32, 38, 48]. Herein, we recommend starting supplemental O_2_ therapy when O_2_ saturation levels are persistently below 92% in room air.

In line with this recommendation, O_2_ saturation must be measured correctly by pulse oximetry. It is recommended to perform gentle nasal aspiration prior to O_2_ saturation measurement, use pediatric probes, and avoid measurement when the baby is moving limbs or experiencing peripheral vasoconstriction [51, 52]. O_2_ saturation measurements should be performed throughout the entire duration of O_2_ supplementation and discontinued in infants with stable clinical improvement and able to feed, and when O_2_ saturation remains steadily above 93% [51, 52]. In infants with comorbidities predisposing to severe respiratory failure, the O_2_ saturation measurements should not be discontinued even after weaning off O_2_ therapy and until the patient is completely stabilized [1, 20–32].

For hospitalized children with bronchiolitis who are not receiving supplemental O_2_, national and international guidelines recommend against continuous pulse oximetry use since pulse oximetry accuracy can be poor, and it correlates weakly with the severity of respiratory distress [1, 20–32, 53]. Accordingly, the educational outreach and audit and feedback strategies, aiming to reduce the continuous pulse oximetry use, resulted in better clinician appropriateness and, consequently, in a deimplementation of continuous pulse oximetry use [51–54].

To minimize handling, humidified O_2_ may be administered by nasal prongs or masks. A heated, humidified, O_2_ therapy has been proposed. By generating significant distending pressure, HFNC can benefit the airway milieu [55]. HFNC can be considered if standard subnasal supplemental O_2_ fails in hypoxic infants and should not be used as a primary treatment modality [38, 55].

In a systematic review and meta-analysis, authors reported that HFNC as respiratory support for children up to 24 months of age with acute bronchiolitis is superior in avoiding treatment failure compared to the standard O_2_ therapy [56–59]. A faster improvement in Respiratory Distress Assessment Instrument (RDAI), respiratory rate, and O_2_ saturation than standard dry O_2_ therapy has been reported over time [60].

 In general, the hypoxaemia is treated with low-flow administered via nasal prongs at ceiling rates of up to 2–3 L/min or face mask at ceiling rates of up to 15 L/min [38].

It has been reported that an initial high-flow rate of nearly 2 L/kg/min meets patients' flow demands and improves respiratory mechanics and breathing effort [61]. A prospective, observational and analytical study showed that an initial flow of 15 L/min is associated with a faster improvement of respiratory rate and a lower treatment failure rate [62].

O_2_ therapy might be discontinued for babies with O_2_ saturation levels equal to or greater than 93-94% in room air, with minimal respiratory distress and adequate feeding [1, 20–32].

On the contrary, patients who do not respond within the first hour of HFNC treatment commonly require ICU admission [63]. However, to date, no definitive conclusions can be formulated on the effects of HFNC on hospitalization days, rates of ICU admission or intubation, duration of stay in the ICU, duration of O_2_ therapy, and clinical progression [57, 58, 64].

In addition to HFNC, nasal CPAP is also increasingly used as a modality of non-invasive respiratory support for infants with acute, moderate-to-severe bronchiolitis. Thanks to the positive end-expiratory pressure (PEEP) generated, CPAP, delivered by nasal prongs or helmet, increases the positive end-expiratory pressure, counteracts airway resistance, and prevents atelectasis [65, 66]. Moreover, CPAP appeared more efficient than HFNC for initial respiratory support in infants with moderate-to-severe bronchiolitis hospitalized in a pediatric ICU (PICU) [57]. However, the current evidence about its use in patients affected by bronchiolitis needs yet to be validated by high-quality *randomized controlled clinical trials (RCTs)* [67–69].

**Fig. 2:**
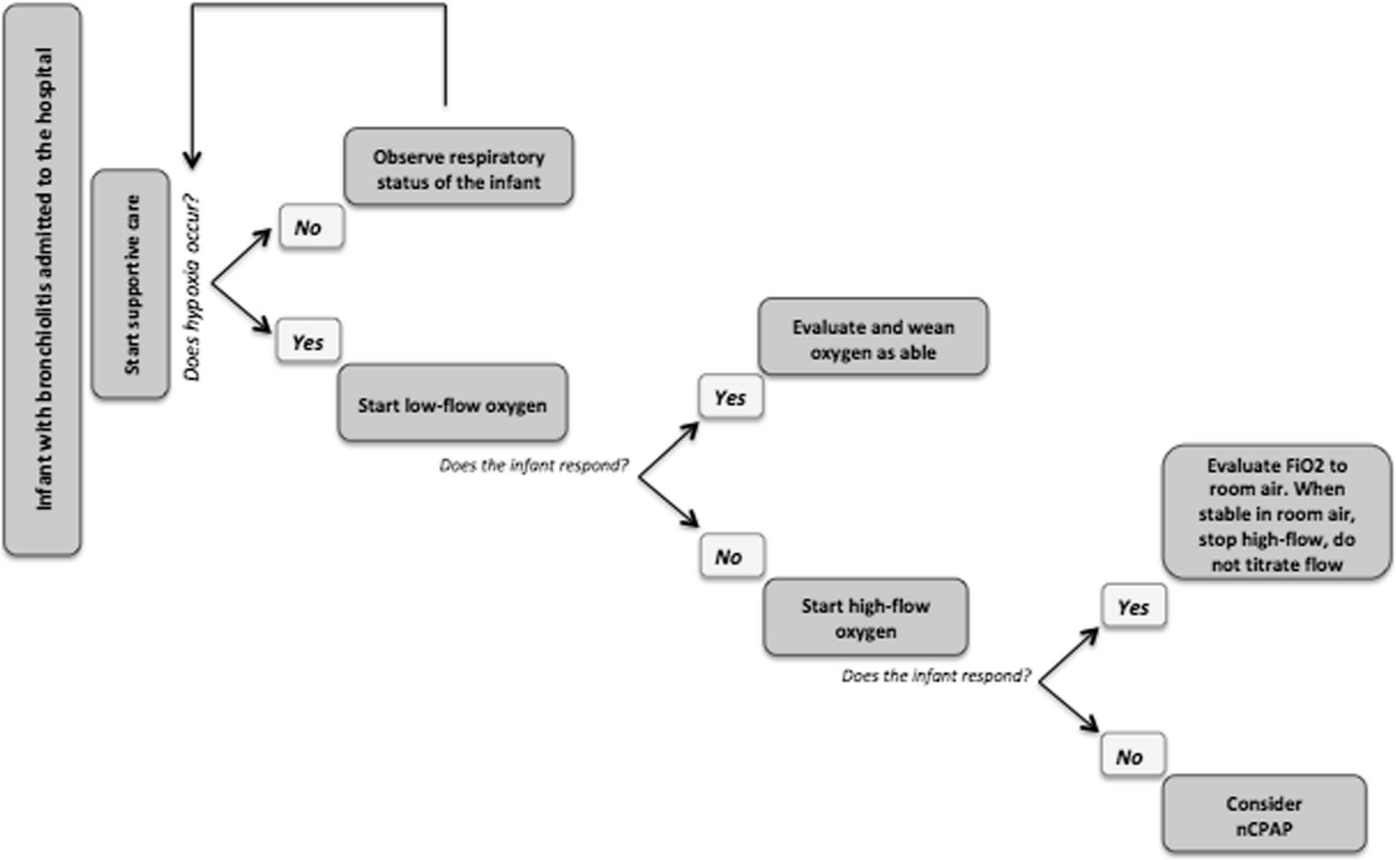
Proposed approach to the use of non-invasive respiratory support in infants with bronchiolitis. FiO_2_: fraction of inspired oxygen; nCPAP: nasal Continuous Positive Airway Pressure

A proposed approach to non-invasive respiratory support in infants with bronchiolitis is reported in Fig. 2.

##### Feeding and hydration

*Recommendations:* Adequate feeding and hydration are recommended in treating bronchiolitis since respiratory distress in infants with bronchiolitis can negatively affect the hydration status (Evidence Quality: A; Recommendation Strength: Strong Recommendation).

Breastfeeding or feeding with a baby bottle should be encouraged for all infants with bronchiolitis, even when receiving HFNC [70]. Frequent and small feeds are supported to prevent the risk of inhalation [1].

Guidelines recommend nasogastric feeding or intravenous fluids for patients who cannot tolerate oral feeding (Evidence Quality: X; Recommendation Strength: Strong Recommendation) [1].

Two strategies can be adopted using enteral hydration: continuous feeding or bolus. Although the continuous feeding strategy appears associated with a higher proportion of ICU admissions, it does not correlate with aspiration events. No significant difference in length of stay was found between continuous feeding and bolus for hospitalized infants with bronchiolitis [71]. Enteral feeding also appeared safe in children with bronchiolitis who received HFNC [72]. Intravenous fluids are recommended for infants who cannot tolerate oral feeding [1, 48]. The use of isotonic fluids is recommended as a significant risk for hyponatremia has been correlated with the use of hypotonic fluids [73].

#### Evidence is equivocal for which treatment?

##### Nebulized hypertonic saline solution

*Recommendations:* There is not enough evidence to routinely recommend nebulized hypertonic 3% saline solution for acute bronchiolitis (Evidence Quality: B; Recommendation Strength: Moderate Recommendation).

The previous national consensus recommended using nebulized hypertonic 3% saline solution in treating bronchiolitis [1].

However, controversial data supports the use of the hypertonic saline solution in infants with bronchiolitis. While several RCTs enrolling patients with moderate-to-severe bronchiolitis showed that the administration of nebulized hypertonic saline solution, alone or in addition to other therapies, did not provide any clinical benefit and rather caused more frequently mild adverse events [74–79], other RCTs and systematic reviews reported that nebulized 3% hypertonic solution appeared safe and more effective in improving clinical severity compared to 0.9% saline solution [80–85].

Because the more recent trials and systematic reviews [48] have shown unclear benefits of the nebulized hypertonic solution compared with the older recommendations [1], we are now updating our recommendation and state that there is currently not enough evidence to routinely recommend this treatment for acute bronchiolitis.

#### Which are treatment not recommended based on evidence?

##### Deep nasal aspiration

*Recommendations:* A deep airway aspiration is not recommended as it is associated with increased length of stay and mechanical trauma in infants (Evidence Quality: B; Recommendation Strength: Moderate Recommendation).

However, more recent studies showed that infants with bronchiolitis tolerated nasal and nasopharyngeal suctioning techniques without adverse short-term effects (Evidence Quality: B; Recommendation Strength: Moderate Recommendation) [86–88].

##### Chest physiotherapy

*Recommendations:* Chest physiotherapy as standard clinical practice for hospitalized infants with bronchiolitis cannot be recommended (Evidence Quality: A; Recommendation Strength: Moderate Recommendation).

In line with the previous guidelines [1], a Cochrane review, including 12 RCTs (1249 participants), concluded that none of the chest physiotherapy techniques (conventional, slow passive expiratory techniques or forced expiratory techniques) showed a reduction in the severity of bronchiolitis [89].

Recently, an RCT in ninety-one non-hospitalized infants (mean age 7.9 ± 2.6 months) with mild to moderate bronchiolitis showed that high-frequency chest wall compression (HFCWC) was effective and safe in decreasing the severity of respiratory symptoms [90].

##### Inhaled bronchodilators

*Recommendations:* Clinicians should not administer salbutamol (albuterol) to infants with a diagnosis of bronchiolitis, as salbutamol does not improve O_2_ saturation, duration of symptoms or length of hospital stay, and there is a potential risk of harm (Evidence Quality: B; Recommendation Strength: Strong Recommendation) [91–96].

Accordingly, Italian Medicines Agency (AIFA) does not support using salbutamol in children younger than 2 years of age [96]. Therefore, a single therapeutic trial with salbutamol by aerosol should no longer be considered [1].

##### Nebulized adrenaline

*Recommendations:* Due to the lack of studies, short duration of action, and potential adverse effects, nebulized adrenaline is not recommended (Evidence Quality: B; Recommendation Strength: Strong Recommendation).

However, in a recent, open-labelled, quasi-randomized clinical trial, performed on 34 children with moderate bronchiolitis, the authors reported that nebulized epinephrine as a first-line medication compared to nebulized hypertonic solution significantly reduced the length of hospital stay [97].

More recently, a study showed that nebulized epinephrine, in addition to systemic corticosteroids, was significantly more effective in reducing ventilatory support in infants with severe bronchiolitis and admitted to ICU than standard care [98].

Further studies are needed to evaluate the early use of nebulized adrenaline, as international guidelines have formulated controversial recommendations [48].

##### Nebulized and systemic steroids

*Recommendations:* Using nebulized and systemic corticosteroids alone or in combination with other therapies (epinephrine or bronchodilators) in treating acute bronchiolitis is not recommended (Evidence Quality: A; Recommendation Strength: Strong Recommendation).

Both treatments do not prevent hospital admission and do not improve short- and long-term outcomes in patients with bronchiolitis or the length of hospital stay. Changes in timing, dosage or duration of treatment do not influence the effects of nebulized and systemic steroids [1, 20, 95, 99–104].

Nevertheless, a minority of guidelines suggest using systemic corticosteroids in exceptional circumstances, such as severe bronchiolitis admitted to ICU [48].

##### Antibiotics

*Recommendations:* The use of antibiotics in bronchiolitis is not recommended except in cases with a strong suspicion or clear evidence of a secondary bacterial infection (Evidence Quality: B; Recommendation Strength: Strong Recommendation).

There is no data supporting macrolides' benefits since they failed to exert an antiviral effect, decrease the length of hospital stay, or supplemental oxygen use in children with bronchiolitis. In addition, the use of macrolides is also useless for most bacterial infections since these are resistant in at least 40% of cases [105, 106].

Further studies are required to assess the short- and long-term clinical outcomes following the use of azithromycin in children with bronchiolitis [1, 20, 32, 48, 93, 107].

##### Other therapies

*Recommendations:* The administration of antivirals (ribavirin), montelukast, DNase, inhaled furosemide, inhaled ipratropium bromide, magnesium sulfate, helium, surfactant, and methylxanthine in children with acute bronchiolitis are not supported by the current evidence (Evidence Quality: B; Recommendation Strength: Strong Recommendation) [1, 20–32, 38, 48].

### What criteria should be used for safe discharge?

*Recommendations:* Except for different O_2_ saturation thresholds recommended for guiding hospital discharge, international guidelines are unanimous on the following criteria for discharge: 1) protracted autonomy from any respiratory support and O_2_ saturation levels greater than 93% in room air; 2) patient clinically stable; 3) adequate oral intake of fluids and feeds (>75% of usual volume); 4) family unit able in coping, spotting red flag symptoms, monitoring and administrating therapy at home; 5) availability of pediatric health care assistance locally if required (Evidence Quality: B; Recommendation Strength: Strong Recommendation) [1, 20–32, 38, 48].

### How can we prevent bronchiolitis?

*Recommendations:* Because of the lack of effective treatment, reduction of morbidity from RSV and other viral infections causing bronchiolitis must rely on preventive measures (Evidence Quality: B; Recommendation Strength: Strong Recommendation).

#### Environmental prophylaxis

Before and after direct contact with the patient, after contact with potentially contaminated objects, all people should disinfect hands (Evidence Quality: B; Recommendation Strength: Strong Recommendation). Cleaning of solid surfaces using water and disinfectants or sodium hypochlorite is strongly supported (Evidence Quality: B; Recommendation Strength: Strong Recommendation).

Exposure to tobacco smoke must be discouraged (Evidence Quality: B; Recommendation Strength: Strong Recommendation) [108, 109].

Exclusive breastfeeding for at least six months should be encouraged to decrease the morbidity of respiratory infections (Evidence Quality: B; Recommendation Strength: Strong Recommendation) [1, 20–32, 109–111].

Since viruses are easily spread by horizontal transmission, via saliva droplets, and through contact with contaminated objects and surfaces, environmental prophylaxis must be carried out to reduce the diffusion of respiratory viruses [1, 20–32]. Frequent handwashing and decontamination of hands using alcohol solutions by parents or caregivers and other household contacts are recommended. Sharing kitchen utensils and personal effects must be avoided [1, 20–32].

Visitors and contacts should be avoided or limited, especially with subjects suffering from respiratory infection symptoms [1, 20–32].

Table 7 summarizes the encouraging behaviours of parents or caregivers caring for an infant with bronchiolitis.

**Table 7:** Parent behaviour: caring for an infant with bronchiolitis

*How parents can protect their babies from bronchiolitis*	
• Exclusive breastfeeding for at least six months should be encouraged to decrease the morbidity of respiratory infections • Frequent handwashing and decontamination of hands using alcohol solutions by parents or caregivers and other household contacts • Use the face mask in case of a cold when approaching the baby. If you have a cold, refrain from kissing the baby and avoid touching his/her face. • Visitors and contacts, especially with subjects suffering from respiratory infection symptoms, should be avoided or limited. • Exposure to tobacco smoke must be strongly discouraged • Ask the pediatrician for monoclonal antibodies use for the prevention of RSV infections, if indicated	
*When to ask for primary care paediatricians*	
• Respiratory distress suggested by cough; dyspnea; polypnea; increased respiratory effort manifested as nasal flaring, grunting, use of accessory muscles or intercostal and/or subcostal chest wall retractions; apnea; skin colour changes. • Feeding difficulties: intake of fluids and feeds < 50% than usual; and signs of dehydration: dry mouth, fewer wet diapers, crying without producing tears. • Poor responsiveness, lethargy and generally toxic appearance, especially in infants younger than 3 months of age	
*Which supportive therapies to administer*	
• A gentle, superficial and reasonably frequent nasal aspiration, especially before feeding • Fractionated and frequent (2–3 hourly) meals	

#### Environmental prophylaxis in healthcare settings

Stethoscope cleaning practices should be followed to prevent the transmission of hospital-acquired infections. Alcohol-based disinfectant is an efficacious agent for cleaning stethoscopes (Evidence Quality: B; Recommendation Strength: Strong Recommendation) [1, 20–32].

The following indications have been proposed to minimize nosocomial RSV infection: 1) use of rapid tests to identify RSV-positive patients for cohorting, to decrease antibiotic use and for epidemiological surveillance; 2) use of disposable gloves for healthcare providers who are in contact with the patient; 3) use of barrier devices in case of manoeuvers which put into contact with respiratory secretions (feeding, airway aspiration, aerosol therapy).

#### Pharmacological prophylaxis

The Italian Society of Neonatology (SIN) [112] recommends palivizumab prophylaxis during RSV season (November-March) for infants of gestational age < 29 weeks and age <12 months at the beginning of the epidemic season (Evidence Quality: A; Recommendation Strength: Strong Recommendation) and for infants of 29-35 weeks gestational age and age < 6 months in the presence of risk factors (Evidence Quality: B; Recommendation Strength: moderate recommendation). The use of Palivizumab in preterm infants born after 29 weeks of gestation remains controversial due its high costs. Palivizumab is also recommended for infants diagnosed with BPD (during their first year of life and during the second year of life in children who require medical therapy) and infants with hemodynamically significant congenital heart disease who are < 12 months of age at the beginning of the epidemic season. In addition, immunoprophylaxis can be considered for infants with cystic fibrosis, Down syndrome, congenital diaphragmatic hernia, neuromuscular diseases and immunodeficiency (Evidence Quality: B; Recommendation Strength: moderate recommendation) [112–114].

Pharmacological prophylaxis of bronchiolitis is based on the prevention of RSV infection. Reducing the global burden of RSV-related illness is considered a global health priority, and developing prevention strategies is a key priority for the WHO [115]. The only currently licensed immunoprophylaxis for RSV is the monoclonal antibody (mAb) palivizumab produced by recombinant DNA technology and targeting the fusion (F) protein of the virus. Evidence has shown that palivizumab, approved in 1999, effectively reduces hospitalization and prevents lower respiratory tract infections in preterm infants [113–115]. It is administered via intramuscular injection once each month during the RSV season for five doses (i.e. 15 mg/kg).

Similar recommendations to those proposed by SIN have been reached by a recent consensus produced by experts from Europe, Canada and Israel [116].

New long-acting mAbs will soon be available on the market. A newer mAb, Nirsevimab, has been recently approved by the European Medicines Agency [117]. It offers protection of 5 months, enabling coverage of the entire RSV season with a single intramuscular dose. In a recent study, nirsevimab reduced medically attended RSV-associated LRTI by 70% and RSV hospitalization by 78% versus placebo in healthy preterm infants [118]. Another long-acting mAb in development is MK-1654 [119]. These new long-acting mAbs represent a new effective strategy for protecting of all infants entering their first RSV season [120]. Another strategy under development for preventing RSV infection is passive immunization through maternal vaccination and active immunization of infants older than 6 months [121].

## Long-term consequences of bronchiolitis

There is growing evidence showing an unequivocal relationship between early-in-life RSV infection and subsequent wheezing illness into childhood and adolescence, suggesting a possible role of RSV in the inception of asthma [122–124]. Hospitalization for bronchiolitis and the severity of the first episode are the main risk factors for developing subsequent wheezing [109]. However, it is unclear whether RSV infection is a causal factor, a marker of susceptibility to respiratory illness, or both. More research is needed to elucidate the pathophysiological mechanisms through which bronchiolitis is associated with recurrent wheezing/asthma [123–125].

## Conclusions

Some aspects of the optimal diagnostic and therapeutic management of viral bronchiolitis are still under debate. Although the scientific community has underlined the importance of avoiding unnecessary and futile diagnostic procedures and therapies, this advice has been ignored frequently. Currently, there is no unequivocal agreement regarding the diagnosis of bronchiolitis, risk factors for severe disease, hospital admission criteria, treatment, and discharge criteria [126–128]. Aiming to clarify and assist clinicians in decision-making for the management of children with viral bronchiolitis, we updated the national guidelines after carefully considering the best evidence available.

- The diagnosis of bronchiolitis is based on the clinical history and physical examination. Laboratory and instrumental investigations are not routinely recommended. Most children with acute bronchiolitis may be adequately managed in the outpatient setting by primary care paediatricians, parents or caregivers able to provide assistance and monitoring.
- The decision to admit to the hospital should be based on clinical conditions suggesting a moderate to severe bronchiolitis (Table 3), O_2_ saturation levels persistently lower than 92%, ability to maintain adequate hydration, and uncertainty over the diagnosis of bronchiolitis (Table 6).
- Since a specific etiological treatment is not available, bronchiolitis therapy includes general supportive management and pharmacological treatment to control pulmonary and systemic clinical symptoms. Gentle nasal aspiration, O_2_ therapy, adequate feeding and hydration remain the cornerstones for the management of bronchiolitis. Deep nasal aspiration, chest physiotherapy, inhaled bronchodilators, nebulized adrenaline, nebulized and systemic corticosteroids, antibiotics, and other therapies are not routinely recommended in treating bronchiolitis.
- When supplementary O_2_ is indicated, HFNC should not be used as a primary treatment modality but considered if standard subnasal supplemental O_2_ fails in hypoxic infants. If respiratory failure requiring ventilatory support (CPAP), apnea with desaturation, and severe impairment of general conditions occur, the baby affected by acute bronchiolitis must be referred to the ICU.
- Since there is no vaccine against RSV (i.e., the main aetiologic agent of bronchiolitis), environmental prophylaxis is crucial in preventing and limiting bronchiolitis spreading. Pharmacological immunoprophylaxis (Palivizumab) has proven beneficial to populations at increased risk for RSV infection–related complications.
- Globally, evidence suggests that less treatment is better and promotes supportive rather than interventional therapy. Nevertheless, universal de-implementation of unnecessary care did not occur and remained a major challenge. Issuing bronchiolitis guidelines is not enough to obtain adherence and clinically significant changes in clinical practice [126, 127].
- Well-prepared implementation strategies to standardize care and improve the quality of care are needed, such as educational interventions and audit cycles for clinicians (working in hospitals, primary care paediatricians) and nursing staff, to promote adherence to guidelines and discourage wrong attitudes, including the use of diagnostic procedures and non-evidence based therapeutic approaches. By sharing the same planning of the intervention, primary care paediatricians and emergency department physicians may feel more supported in adopting a shared approach, thereby significantly reducing overtreatment and nonadherence to guidelines [128].
- In the future, taking advantage of the use of electronic medical records, it may be reasonable to promote the adoption of a “warning signal” when a prescribed therapy might not be appropriate for a patient diagnosed with bronchiolitis.
- In parallel, educating parents with a “wait and see” approach could allow symptoms to improve spontaneously and reduce patient pressure, contributing to inappropriate prescriptions. In this regard, the publication and diffusion of educational materials could implement choosing wisely recommendations and change non-evidence-based clinical practice. Moreover, it would increase the awareness that the inappropriate prescription of drugs is not only useless but also potentially harmful, as associated with bacterial resistance and risk of severe patient status, lower O_2_ saturation, fever, and tachycardia induced by inhaled salbutamol [96, 129–131].
- Similarly to monitoring RSV circulation and activity in the community, creating a system able to reflect, in near real-time, the timing and the community-based intensity of RSV circulation could be helpful. This intervention could provide pathogen surveillance and, consequently, detect early viral warning of activity and threshold of public health intervention, monitor the current healthcare behaviour among different healthcare institutions, and improve the intervention measures, thus, the public health response.

This national guideline will be updated as new evidence arises in this field to report new evidence and promote the proper management of infants with viral bronchiolitis.

## Supplementary Information

**Additional file 1: Appendix 1.** Our research has been conducted employing PubMed, EMBASE, and Global Health databases. On these websites, we searched for articles from January 1st, 2014, to 1 April 2022, using key terms related to bronchiolitis in pediatric population. **Appendix 2.** AGREE II instrument domains of quality assessment.

## Data Availability

Not applicable

## Abbreviations

AIFA: Italian Medicines Agency
BPD: Bronchopulmonary dysplasia
CPAP: Continuous positive airway pressure
COVID-19: Coronavirus disease of 2019
GRADE: Grading of Recommendations, Assessment, Development and Evaluations
HFNC: High flow nasal cannula
HFCWC: High-frequency chest wall compression
ICU: Intensive care unit
MPV: Metapneumovirus
O2: Oxygen
PEEP: Positive end-expiratory pressure
PICU: Pediatric intensive care unit
RCTs: Randomized controlled clinical trials
RDAI: Respiratory Distress Assessment Instrument
RSV: Respiratory Syncytial Virus
RT-PCR: Reverse transcription polymerase chain reaction (RT-PCR)
RV: Rhinovirus
WHO: World Health Organization
