# Supplementary material for: UPDATE - 2022 Italian guidelines on the management of bronchiolitis in infants
Source: Ital J Pediatr. 2023 Feb 10;49:19. doi: 10.1186/s13052-022-01392-6 (PMC9912214; doi:10.1186/s13052-022-01392-6)
Supplement: Supplementary file 1 — Additional file 1: Appendix 1. Our research has been conducted employing PubMed, EMBASE, and Global Health databases. On these websites, we searched for articles from January 1st, 2014, to 1 April 2022, using key terms related to bronchiolitis in pediatric population. Appendix 2. AGREE II instrument domains of quality assessment. [file 13052_2022_1392_MOESM1_ESM.docx]

**Online Repository**

**Appendixes.**

**Appendix 1.** Our research has been conducted employing PubMed, EMBASE, and Global Health databases. On these websites, we searched for articles from January 1st, 2014, to 1 April 2022, using key terms related to bronchiolitis in pediatric population.

1. Bronchiolitis
2. Acute
3. Viral
4. Newborn
5. Infant
6. Child
7. Children
8. RSV
9. Respiratory Syncytial Virus
10. 1 or 2 or 3 or 4 or 5
11. 1 and 4 or 5 or 6 or 7 or 8 or 9
12. Guideline/ or practice guideline/
13. Guidelines as topic/ or practice guidelines as topic/
14. (guideline* or algorithm* or standard*).ti.ab.
15. “best practice”.ti.ab.
16. 11 or 12 or 14

**Appendix 2.** AGREE II instrument domains of quality assessment

Domain 1 – Scope and Purpose

- The overall objective(s) of the guideline is (are) specifically described
- The health question(s) covered by the guideline is (are) specifically described
- The population (patients, public, etc.) to whom the guideline is meant to apply is specifically described

Domain 2 – Stakeholder Involvement

- The guideline development group includes individuals from all relevant professional groups
- The views and preferences of the target population (patients, public, etc.) have been sought
- The target users of the guideline are clearly defined

Domain 3 – Rigour of Development

- Systematic methods were used to search for evidence
- The criteria for selecting the evidence are clearly described
- The strengths and limitations of the body of evidence are clearly described
- The methods for formulating the recommendations are clearly described
- The health benefits, side effects and risks have been considered in formulating the recommendations
- There is an explicit link between the recommendations and the supporting evidence
- The guidance has been externally reviewed by experts prior to its publication
- A procedure for updating the guideline is provided

Domain 4 – Clarity of Presentation

- The recommendations are specific and unambiguous
- The different options for management of the condition or health issue are clearly presented
- Key recommendations are easily identifiable

Domain 5 – Applicability

- The guideline describes facilitators and barriers to its application
- The guideline provides advice and/or tools on how the recommendations can be put into practice
- The potential resource implications of applying the recommendations have been considered
- The guideline presents monitoring and/or auditing criteria

Domain 6 – Editorial Independence

- The views of the funding body have not influence the content of the guideline
- Competing interests of guideline development group members have been recorded and addressed
